# Correction: ‘The emergence of nonlinear evolutionary trade-offs and the maintenance of genetic polymorphisms’ (2024), by Hulse and Bruns

**DOI:** 10.1098/rsbl.2025.0020

**Published:** 2025-02-19

**Authors:** Samuel V. Hulse, Emily L. Bruns

**Affiliations:** ^1^ University of Maryland, College Park, MD, USA

*Biol. Lett*. **20**, 20240296 (Published online 4 December 2024). (https://doi.org/10.1098/rsbl.2024.0296).

Since the initial publication of our manuscript [1], we found an error in the figure caption for figure 1. In the original caption, the panels are referred to as linear costs (figure 1*a*), decelerating costs (figure 1*b*) and accelerating costs (figure 1*c*). The correct label is instead linear costs (figure 1*a*), accelerating costs (figure 1*b*) and decelerating costs (figure 1*c*). While the figure legend was mislabelled, the inline figure references are correct in our original manuscript. This error does not affect any of our results or discussion.

This has been corrected on the publisher's website.

## References

[1] Hulse SV, Bruns EL. 2024 The emergence of nonlinear evolutionary trade-offs and the maintenance of genetic polymorphisms. Biol. Lett. **20**, 20240296. (10.1098/rsbl.2024.0296) 39626761 PMC11614545

